# Clinicopathological and prognostic characteristics of idiopathic membranous nephropathy with dual antigen positivity

**DOI:** 10.3389/fimmu.2023.1297107

**Published:** 2024-01-05

**Authors:** Lei Yang, Guoqin Wang, Nan Ye, Xiaoyi Xu, Wenrong Cheng, Lijun Sun, Hongrui Dong, Lingqiang Kong, Xiaoyi Zhao, Yanqiu Geng, Hong Cheng

**Affiliations:** ^1^ Beijing Anzhen Hospital, Capital Medical University, Beijing, China; ^2^ Division of Nephrology, Affiliated Hospital of Chifeng University, Neimenggu, China; ^3^ Division of Nephrology, The Third Medical Center, Chinese PLA General Hospital, Beijing, China

**Keywords:** idiopathic membranous nephropathy, PLA2R, THSD7A, NELL-1, dual antigen

## Abstract

**Background:**

Idiopathic membranous nephropathy (IMN) is the most common pathological type in adults with nephrotic syndrome. Many target antigens have been discovered. However, dual antigen-positive IMN patients are very rare, with only a few such cases being briefly described in various studies. There is no specific study on the clinicopathological and prognostic characteristics of dual antigen-positive IMN patients, and the disease characteristics of such patients remain unclear.

**Methods:**

Immunohistochemical staining of PLA2R, THSD7A, and NELL-1 was conducted on kidney tissue samples obtained from patients diagnosed with IMN. Simultaneously, the presence of corresponding serum antibodies was determined. Patients exhibiting positivity for dual antigens were included in the study, identified either through tissue staining or serum antibody detection. We retrospectively collected their clinical, pathological, and follow-up data and measured their serum antibody levels at multiple time points. Additionally, the same type of dual antigen-positive IMN cases reported in the literature were reviewed to extract clinical, pathological, and prognostic information. We compared the data for all of the above dual antigen-positive and PLA2R single-positive IMN cases at our center.

**Results:**

We identified 6 IMN patients with dual antigen positivity at our center, approximately 0.7% of whole MN series; the previous literature reports 43 IMN patients with dual antigen positivity, the proportion ranged from 0.2% to 2.8%. The IgG1 positivity rate in the renal tissue of the dual antigen-positive patients at our center was significantly lower than that of dual antigen-positive patients previously reported (16.7% vs. 100.0%, p=0.015), but there was no significant difference in clinical or prognostic aspects. Patients with dual antigen positivity reported at our center and in the literature were combined and compared with PLA2R single-positive IMN reported at our center. Compared with PLA2R single-positive IMN patients, dual antigen-positive IMN patients had a higher renal tissue IgG1 positivity rate (58.3% vs. 22.3%, p=0.016), and the time required to achieve remission was longer [13.5 (3.3,35.0) vs. 3.0 (1.0,8.0), p=0.052]. Overall, The changes in urine protein were consistent with the changes in serum PLA2R antibody levels in dual antigen-positive IMN patients.

**Conclusions:**

For patients with primary membranous nephropathy who did not attain remission following prolonged treatment, multiple target antigen staining should still be actively performed, even with positivity for the PLA2R target antigen.

## 1 Introduction

Idiopathic membranous nephropathy (IMN) is an immune-mediated primary glomerular disease and the most common pathological type in adults with nephrotic syndrome (1). Research has confirmed that the main pathogenesis of IMN is specific binding of circulating antibodies to target antigens on the glomerular basement membrane to form immune complexes that are deposited in the subepithelial area, activating the complement cascade, causing podocyte damage, and ultimately leading to proteinuria (2). Since M-type anti-phospholipase A2 receptor (PLA2R), the first specific target antigen in adult IMN, was discovered in 2009 (3) research on IMN target antigens has been growing rapidly. Indeed, many IMN target antigens have been discovered over the past decade, including thrombospondin type 1 domain containing 7A (THSD7A) and neuroepidermal growth factor-like type 1 protein (NELL-1) (4, 5). However, the vast majority of IMN patients reported thus far are single antigen-positive; in contrast, dual antigen-positive IMN patients are very rare, with only a few such cases being briefly described in various studies (6–8). There is no specific study on the clinicopathological and prognostic characteristics of dual antigen-positive IMN patients, and the disease characteristics of such patients remain unclear.

Here, we provide a detailed description of the clinical pathological characteristics and prognosis of dual antigen-positive IMN patients at our center. In addition, we reviewed previous literature on dual antigen-positive IMN cases, extracted patient information, and compared the clinical and pathological data of dual antigen-positive IMN cases reported thus far with those of PLA2R single-positive IMN cases in an effort to help clinicians further understand this rare dual antigen-positive IMN.

## 2 Materials and methods

### 2.1 Information collection of dual antigen-positive IMN and PLA2R single-positive IMN patients at our center

We continuously reviewed patients diagnosed with IMN by renal biopsy in the Department of Nephrology, Beijing Anzhen Hospital, Capital Medical University from 2015 to 2019. By staining of PLA2R, THSD7A, NELL-1 antigen in kidney tissue and detection of corresponding serum antibodies, we screened the dual antigen- positive IMN and PLA2R single-positive IMN, diagnosed by positive tissue staining or positive serum antibodies. Their baseline clinical, pathological and prognostic information were retrospectively collected. Follow-up data were obtained by reviewing the medical records and/or from telephone interviews of patients, and they were available on some patients. The treatment and prognostic analyses were performed in patients with complete information on follow-up. The definitions of remission complied with the 2012 Kidney Disease Improving Global Outcomes guideline for glomerular nephropathy (9). Complete remission was defined as urinary protein excretion <0.3 g/d, confirmed by two values at least 1 week apart, accompanied by normal serum albumin and creatinine levels. Partial remission was defined as urinary protein excretion <3.5 g/d and at least a 50% reduction from peak values accompanied by an improvement or normalization of serum albumin and stable serum creatinine levels. Complete remission and partial remission are collectively referred to as remission. Worsening of renal function was defined as a doubling of creatinine.

### 2.2 Immunohistochemical staining of renal tissue in dual antigen-positive IMN patients at our center

Paraffin-embedded kidney tissue (4 μm) was obtained. PLA2R and NELL-1 antigens were retrieved with pH 6 citrate solution combined with trypsin; THSD7A was retrieved with pH 9 EDTA solution. The primary antibodies used were monoclonal rabbit anti-human PLA2R1 antibody (Sigma, HPAO12657) (1:800 dilution), monoclonal rabbit anti-human NELL-1 antibody (Sigma, HPAO51535) (1:400 dilution), and monoclonal rabbit anti-human THSD7A antibody (Sigma, HPAO00923) (1:3000 dilution), which were incubated overnight at 4°C. The secondary antibody, alkaline phosphatase-labeled immunohistochemistry reagent (Max Vision, KIT-5103) or horseradish peroxidase-labeled immunohistochemistry reagent (Max Vision, KIT-5004), was added and incubated at room temperature for 30 minutes. Fast-Red reagent (Zhongshan Jinqiao, ZLI-9042) or DAB reagent (Zhongshan Jinqiao, ZLI-9018) was used for color development. Antigen positive is judged as granular brown staining in the glomerular capillary loops, while the renal tubule and other background staining linear reduction (10).

### 2.3 Other pathological staining and electron microscope examination of renal tissue

The routine fluorescent staining and histochemical staining are carried out in accordance with the standard procedures and the recommended procedures of the reagent specification. Fluorescence intensity criteria: there is no light at both low and high magnification as “-”; negative at low magnification, seemed to be visible at high magnification as “±”; it seems to be visible at low magnification, and blurred at high magnification as “+”; it is obviously visible at low magnification and clearly visible at high magnification as “++”; clearly visible at low magnification and dazzling fluorescence at high magnification as “+++”: dazzling at low magnification and dazzling fluorescence at high magnification as “++++”. Electron microscope examination of renal tissue was delivered to the testing center.

### 2.4 Antibody detection in serum of dual antigen-positive IMN patients at our center

Serum samples were collected on the day of kidney biopsy and stored at -80°C until use. Serum anti-PLA2R antibodies were detected using the ELISA (EUROIMMUN, Germany) method. Serum anti-THSD7A and anti-NELL-1 antibodies were detected using an indirect immunofluorescence assay kit (EUROIMMUN, Germany) according to the standard protocol.

### 2.5 Systematic review of previous reports on IMN patients with dual antigen positivity

We searched for “M-type anti-phospholipase A2 receptor”, “thrombospondin type 1 domain containing 7A”, and “neuroepidermal growth factor-like type 1 protein” in the three major English databases Medline, Embase, and Cochrane Library and the two major Chinese databases Wan fang and CNKI (the specific search strategies are provided in **Supplementary Material**). All literature related to dual antigen-positive IMN was screened as of June 6, 2023, and literature information and clinicopathological and prognostic data of dual antigen-positive patients were extracted.

### 2.6 Statistical methods

The continuous variables of normal distribution were represented by mean ± standard deviation, and the difference between groups was compared by independent sample t test. Continuous variables with non-normal distributions were expressed as median and quartile distances (P25, P75), and non-parametric tests were used to compare group differences. The number and percentage of categorical variables were expressed, and Chi-square test and Fisher exact test were used to compare the differences between groups. SPSS 23.0 software was used for statistical analysis, and bilateral P value <0.05 was considered statistically significant.

## 3 Results

### 3.1 Clinicopathological characteristics and prognosis of dual antigen-positive IMN patients at our center

A total of 827 patients diagnosed with IMN by renal biopsy at our center from 2015 to 2019, with an average age of 48 years old, accounted for 63.6% of males. Through tissue antigen staining and serum antibody detection, 6 patients with dual antigen positivity were screened, including 3 PLA2R- and NELL-1-positive patients and 3 PLA2R- and THSD7A-positive patients. Detection of tissue antigens and serum antibodies in the 6 patients is shown in **Table 1**.

**Table 1A:** Clinicopathological and prognostic characteristics of 6 IMN patients with dual antigen positivity at our center.

		PLA2R		NELL-1		THSD7A	
		tissue antigens	serum antibodies(RU/ml)	tissue antigens	serum antibodies(RU/ml)	tissue antigens	serum antibodies(RU/ml)
PLA2R- and NELL-1-positive							
	MN1	+	12.80	+	–	–	–
	MN2	+	0.00	+	–	–	–
	MN3	+	125.10	+	–	–	–
PLA2R- and THSD7A-positive							
	MN4	+	41.49	–	–	+	–
	MN5	+	0.00	–	–	+	–
	MN6	+	225.50	–	–	+	–

**Table 1B:** Clinicopathological and prognostic characteristics of 6 IMN patients with dual antigen positivity at our center.

Clinical features									Pathological features					
Sex	Age(year)	Diabetes	Tumor	24h-urinary protein (g/24h)	ALB(g/L)	Scr(μmol/L)	eGFR(mL/(min·1.73 m^2^))	ANA	Stage	Grading of renal interstitial injury	Mesangial hyperplasia	IgA	IgG	IgM
M	68	Yes	No	5.17	28.60	59.20	98.75	weak+	II	1	No	–	3+	–
M	46	No	No	3.70	29.30	71.60	106.59	–	I	1	No	–	3+	–
F	65	No	No	5.18	33.50	57.00	93.71	–	II	1	No	–	3+	–
F	77	Yes	No	4.80	15.70	97.40	48.45	–	I	1	Yes	–	2+	–
M	29	No	No	1.23	41.70	93.00	95.23	–	II	0	No	–	2+-3+	–
M	44	No	No	2.40	24.50	71.00	108.47	NA	II	0	No	–	3+	–

**Table 1C:** Clinicopathological and prognostic characteristics of 6 IMN patients with dual antigen positivity at our center.

Pathological features					
IgG1	IgG2	IgG3	IgG4	C3	Electron dense deposit site
3+	–	–	3+	1+-2+	subepithelial
–	–	–	2+	–	subepithelial
–	–	–	2+-3+	2+	subepithelial
–	–	–	2+	–	subepithelial
1+-2+	–	–	2+	–	subepithelial, basement membrane
–	–	–	3+	2+	subepithelial

**Table 1D:** Clinicopathological and prognostic characteristics of 6 IMN patients with dual antigen positivity at our center.

Prognostic characteristics					
Treatment	Remission	Duration of remission (m)	Relapse	Progress of renal function	Duration of follow-up (m)
CsA	Yes	4	Yes	No	70
CsA	Yes	20	No	No	38
GCs, CsA	Yes	42	NA	No	42
CsA	No	–	–	No	48
NA	No	–	–	No	1
GCs, CTX	Yes	36	NA	No	36

PLA2R, M-type anti phospholipase A2 receptor; NELL-1, Neuroepidermal growth factor like type 1 protein; THSD7A, Thrombospondin type 1 domain containing 7A; ALB, serum albumin; Scr, serum creatinine; eGFR, estimated glomerular filtration rate; TG, triglycerides; TCHO, total cholesterol; M, male; F, female; Renal interstitial injury grading: grade 0, no renal interstitial fibrosis; Grade 1, the range of renal interstitial fibrosis was 1-25%; Grade 2, the range of renal interstitial fibrosis was 26-50%; Grade 3, extent of renal interstitial fibrosis > 50%; Immunofluorescence positive, fluorescence intensity ≥2+; CsA, Cyclosporin; MMF, Mycophenolate Mofetil; CTX, Cyclophosphamide; GCs, Glucocorticoids; RTX, Rituximab; NA, not afford.

The clinical data of the 6 patients are shown in **Table 1**. The median age was 55.5 years old, and 4 patients were older than 45 years. Four patients (66.7%) had significant proteinuria, of whom only 3 (50%) presented with nephrotic syndrome (NS); 1 patient (MN4) had a slight increase in blood creatinine. To date, no concomitant tumor diseases have been detected during follow-up of the 6 patients, but the tumor markers of patient MN1 continued to increase during the course of the disease (**Supplementary Figure 1**).

**Figure 1:**
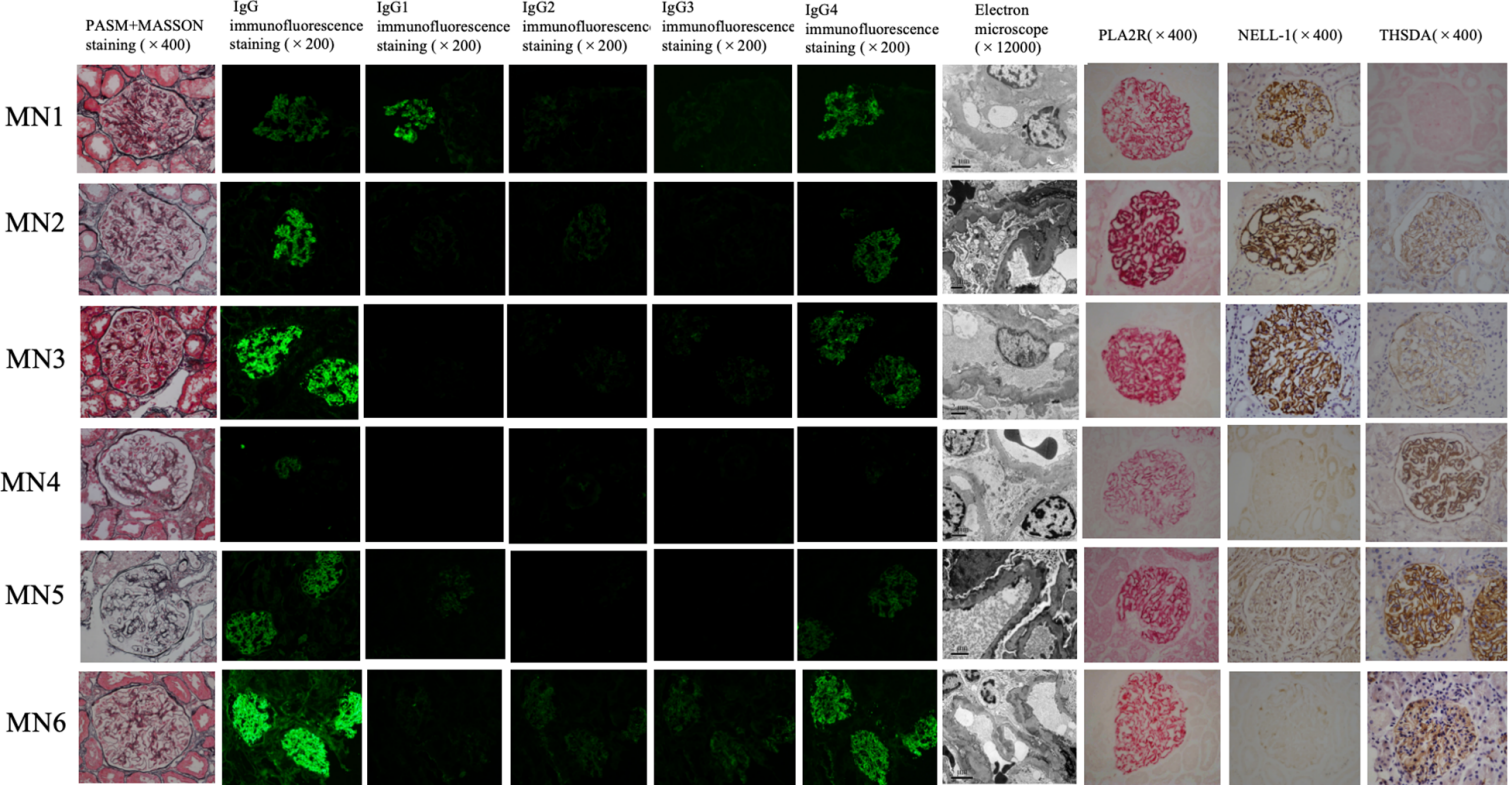
Renal tissue staining of 6 dual antigen-positive IMN patients at our center. From left to right, there are PASM+MASSON staining (×400), IgG immunofluorescence staining (×200), IgG1 immunofluorescence staining (×200), IgG2 immunofluorescence staining (×200), IgG3 immunofluorescence staining (×200), IgG4 immunofluorescence staining (×200), Electron microscope (×12000), PLA2R immunohistochemistry staining (×400), NELL-1 immunohistochemistry staining (×400), THSD7A immunohistochemistry staining in renal tissue. Among the 6 cases,MN1– MN3 are positive for PLA2R and NELL-1 staining; MN4-MN6 are positive for PLA2R and THSD7A staining.

Typical IMN manifestations under light microscopy were observed for these six patients, with all cases showing subepithelial deposition of fuchsinophilic protein. Only one IMN patient with PLA2R and THSD7A positivity (MN4) exhibited mild mesangial cell proliferation and increased mesangial matrix in the glomerulus. Immunofluorescence showed IgG particles deposited along the basement membrane in all 6 patients, without any other immunoglobulin deposits. IgG subclass staining for all 6 patients mainly showed IgG4; only one PLA2R- and NELL-1-positive patient (MN1) showed the same fluorescence intensity as for IgG1 and IgG4. The results of electron microscopy were consistent with those of light microscopy. In all 6 patients, electronic dense matter was found to be deposited in the subepithelial area of basement membrane area of the glomerulus (**Table 1**, **Figure 1**).

The median follow-up time of the 6 patients was 40 months. Except for patient 5, whose treatment regimen we did not track, all patients received immunosuppressive therapy. Four of the patients achieved disease remission after treatment, but only 1 patient (MN1) achieved it within half a year. Of the 2 patients (MN1, MN2) with follow-up data after remission, 1 patient experienced relapse, whereas remission was maintained in the other patient. None of the patients had worsening of renal function during follow-up (**Table 1**).

### 3.2 Literature review and information extraction of previous reports on dual antigen-positive IMN patients

We conducted a systematic review of the literature on previous studies involving double antigen-positive IMN. By June 6, 2023, a total of 11 studies (7, 11–20) involving 43 dual antigen-positive IMN patients, were included (**Supplementary Table 1**, **Supplementary Figure 2**). Of the 43 patients, highly detailed information was available for only 11, and we extracted clinical, pathological, and prognostic information for these 11 patients (**Table 2**) and then performed comparisons with the 6 dual antigen-positive IMN patients at our center (**Supplementary Table 2**).

Table 2Clinicopathological and prognostic characteristics of 11 cases of dual antigen-positive IMN patients in previous studies.

**Table:** 

Study	Case no.	PLA2R		NELL-1		THSD7A		Clinical features								
		tissue antigens	serum antibodies	tissue antigens	serum antibodies	tissue antigens	serum antibodies	sex	Age(year)	Diabetes	Tumor	24h-urinary protein (g/24h)	ALB(g/L)	Scr(μmol/L)	eGFR(mL/(min·1.73 m^2^))	ANA
Hara 2019	1	+	NA	NA	NA	+	NA	M	68	NA	NA	11.6	NA	102.54	NA	NA
Zhang 2019	2	+	+	NA	NA	+	–	M	51	NA	+	NA	NA	NA	NA	NA
Zaghrini 2019	3	+	+	NA	NA	+	+	M	65	NA	NA	6	28	52.16	158	NA
	4	+	+	NA	NA	+	+	M	48	NA	NA	4	25	46.85	192	NA
Subramanian 2020	5	+	NA	NA	NA	+	NA	M	50	NA	NA	NA	NA	NA	NA	NA
	6	–	+	NA	NA	+	NA	M	37	NA	NA	NA	NA	NA	NA	NA
Wanderley 2020	7	+	NA	NA	NA	+	NA	M	46	NA	–	4	15	76.02	NA	–
Xue 2020	8	+	+	NA	NA	+	NA	M	35	–	–	4.9	20.7	124	NA	–
	9	+	NA	NA	NA	+	NA	M	72	NA	–	5.3	20.3	135	NA	–
Yeter 2021	10	+	NA	NA	NA	+	NA	M	18	NA	NA	2.5	NA	53.04	147	NA
Inoue 2023	11	NA	+	NA	+	NA	NA	M	70	NA	–	7.65	22	NA	NA	NA

**Table:** 

Pathological features												Treatment	Prognostic characteristics				
Stage	Grading of renal interstitial injury	Mesangial hyperplasia	IgA	IgG	IgM	IgG1	IgG2	IgG3	IgG4	C3	Electron dense deposit site		Remission	Duration of remission (m)	Relapse	Progress of renal function	Duration of follow-up (m)
NA	NA	–	–	positive	–	2+	–	–	2+	–	NA	GCs, ARB	Yes	32	No	No	32
NA	NA	NA	–	positive	–	positive	NA	NA	positive	positive	NA	FK506	No	–	–	Yes	22
II	1	NA	NA	NA	NA	NA	NA	NA	NA	NA	NA	ACEI/ARB	Yes	3	No	No	40
II	1	NA	NA	NA	NA	NA	NA	NA	NA	NA	NA	ACEI/ARB	No	–	–	No	3
NA	NA	NA	NA	NA	NA	NA	NA	NA	NA	NA	NA	NA	NA	NA	NA	NA	NA
NA	NA	NA	NA	NA	NA	NA	NA	NA	NA	NA	NA	NA	NA	NA	NA	NA	NA
II	NA	+	positive	positive	NA	positive	NA	NA	positive	positive	subepithelial, mesangial region	ACEI/ARB	No	–	–	No	2
II	1	NA	–	3+	–	2+	–	–	3+	2+	subepithelial	GCs, CTX	Yes	7	No	NA	18
II	1	NA	–	2+	–	2+	–	–	3+	2+	subepithelial	GCs, CTX	NA	NA	NA	NA	NA
NA	0	+	NA	NA	NA	NA	NA	NA	positive	NA	NA	GCs, CsA, MMF,RTX	No	NA	NA	Yes	40
I	NA	–	–	2+	–	2+	–	–	–	–	subepithelial	GCs	Yes	1	No	NA	18

PLA2R, M-type anti phospholipase A2 receptor; NELL-1, Neuroepidermal growth factor like type 1 protein; THSD7A, Thrombospondin type 1 domain containing 7A; ALB, serum albumin; Scr, serum creatinine; eGFR, estimated glomerular filtration rate; TG, triglycerides; TCHO, total cholesterol; M, male; F, female; Renal interstitial injury grading: grade 0, no renal interstitial fibrosis; Grade 1, the range of renal interstitial fibrosis was 1-25%; Grade 2, the range of renal interstitial fibrosis was 26-50%; Grade 3, extent of renal interstitial fibrosis > 50%; Immunofluorescence positive, fluorescence intensity ≥2+; Positive staining in fluorescent staining that did not describe the fluorescence intensity was indicated by us as “positive”; CsA, Cyclosporin; MMF, Mycophenolate Mofetil; CTX, Cyclophosphamide; GCs, Glucocorticoids; RTX, Rituximab; NA, not afford.

### 3.3 Comparison of clinicopathological features and prognosis between dual antigen-positive IMN patients and PLA2R single-positive IMN patients

To explore the difference between dual antigen-positive IMN and PLA2R single-positive IMN, we compared the two groups of patients. However, because the number of PLA2R single-positive IMN at our center is much larger than the number of dual antigen-positive IMN in the same time period, we only continuously enrolled 141 patients with PLA2R single-positive IMN within one year (2018-2019)of this time period. The comparison of clinicopathology and prognosis of the two groups at our center is shown in **Supplementary Tables 3, 4**. There was no significant difference in clinicopathology and prognosis between the two groups. Since the number of IMN patients with dual antigen positivity is small, meanwhile, we compared the baseline clinical and pathological data of all known dual antigen-positive IMN patients, including the 6 cases from our center and the 11 cases in the literature with complete clinical and pathological data, with 141 PLA2R single-positive patients at our center (**Table 3**). The results showed that the dual antigen-positive patients had a higher IgG1-positive rate in renal tissue than the PLA2R single-positive patients (58.3% vs. 22.3%, p=0.016). We also conducted a meta-analysis of baseline urine protein and serum albumin comparison results between 17 dual antigen-positive patients and the 141 PLA2R single-positive patients (**Supplementary Figures 3, 4**), with no significant difference in baseline urine protein and blood albumin between the two groups. Prognosis comparison between the two groups showed a longer time to achieve remission in the patients with dual antigen positivity than in those with PLA2R single positivity, even though this difference did not reach statistical significance (p=0.052) (**Table 4**).

**Table 3:** Comparison of clinical and pathologic data between all dual antigen-positive IMN patients and PLA2R single-positive IMN patients at our center.

Characteristics	IMN with dual antigen positivity(n=17)	IMN with PLA2R single positivity(n=141)	P-value
Male(%)	15 (88.2)	91 (64.5)	0.050
Age(year)	50.0 (40.5, 68.0)	53.0 (44.3, 63.0)	0.910
Hypertension(%) (n=152)	6 (54.5)	80 (56.7)	1.000
Diabetes(%) (n=147)	2 (28.6)	22 (15.7)	0.708
24h-urinary protein(g/24h)	4.9 (3.4, 5.5)	4.9 (3.0, 8.3)	0.510
ALB(g/L)	24.8 (20.4, 29.1)	25.9 (22.1, 32.1)	0.404
Scr(μmol/L)	71.6 (55.0, 99.9)	68.0 (57.1, 79.1)	0.439
eGFR(mL/(min·1.73 m^2^))	106.6 (94.5, 152.5)	102.3 (86.7, 116.0)	0.340
Pathological stage(n=151)			0.264
I	3 (25.0)	35 (26.0)	
II	9 (75.0)	75 (55.5)	
III	0	25 (18.5)	
Crescent body(%)(n=152)	0	4 (2.8)	1.000
Renal interstitial injury grading(n=151)			0.257
0	3 (27.3)	16 (11.4)	
1	8 (72.7)	97 (69.3)	
2	0	24 (17.1)	
3	0	3 (2.1)	
IgG positive(%)(n=153)	12 (100)	140 (99.3)	1.000
IgG1 positive(%)(n=151)	7 (58.3)	31 (22.3)	0.016
IgG2 positive(n=149)	0	8 (5.8)	1.000
IgG3 positive(%)(n=148)	0	9 (6.5)	1.000
IgG4 positive(%)(n=152)	12 (92.3)	117 (84.2)	0.705
Simultaneous positivity of IgG1 and IgG4 (%)	6 (50.0)	29 (20.9)	0.053
IgM positive(%)(n=152)	0	17 (12.1)	0.468
IgA positive(%)(n=153)	1 (8.3)	21 (14.9)	0.847
C3 positive(%)(n=153)	6 (50)	108 (76.6)	0.092
C1q positive(%)(n=152)	0	4 (2.8)	1.000

ALB, serum albumin; Scr, serum creatinine; eGFR, estimated glomerular filtration rate.

**Table 4:** Comparison of prognosis between all dual antigen-positive IMN patients and PLA2R single-positive IMN patients at our center.

Characteristics	IMN with dual antigen positivity(n=17)	IMN with PLA2R single positivity(n=62)	P-value
Remission(%)(n=76)	8 (57.1)	43 (69.4)	0.530
Duration of remission(m)	13.5 (3.3, 35.0)	3.0 (1.0, 8.0)	0.052
Remission within six months(%)(n=54)	3 (27.3)	23 (53.5)	0.120
Worsening of renal function(%)(n=74)	2 (16.7)	6 (9.7)	0.608
Immunosuppressive therapy(%)(n=74)	11(78.6)	46(76.7)	1.000

### 3.4 Follow-up of serum antibodies in dual antigen-positive IMN patients

**Figure 2:**
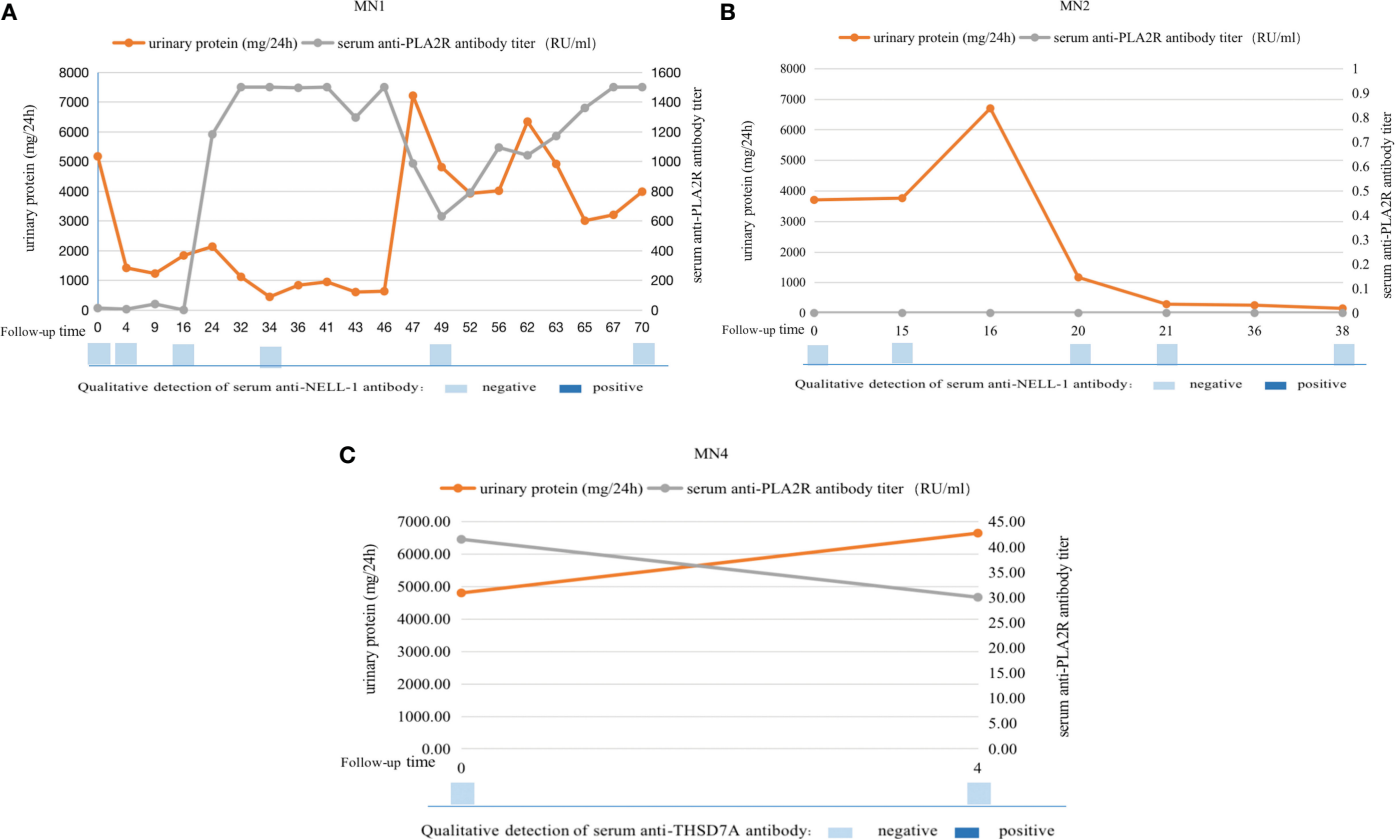
Serum antibody levels were measured at multiple nodes of disease change for three of the patients at our center. MN1 patient (PLA2R- and NELL-1-positive) achieved remission after treatment, but relapsed 46 months later. His serum anti-PLA2R antibody titers were consistent with changes in urine protein, but his serum anti-NELL-1 antibody remained negative throughout the disease course **(A)**; MN2 patient (PLA2R- and NELL-1-positive) achieved complete remission after treatment and there was no recurrence thereafter. His serum anti-PLA2R antibody titers continued to be 0R/ml and serum anti-NELL-1 antibodies continued to be negative **(B)**; MN4 patient (PLA2R- and THSD7A-positive) did not improve after 4 months of treatment, with a slight decrease in serum anti-PLA2R antibody titers and continuous negative serum anti-THSD7A antibodies **(C)**.

Serum antibody levels were measured at multiple nodes of disease change for three of the patients at our center (MN1, MN2, and MN4) (**Figure 2**). The condition of MN1 was protracted, starting with NS. After 4 months of treatment, partial remission was achieved, and the serum anti-PLA2R antibody titer gradually decreased. After 4 years, relapse of NS occurred, and the serum anti-PLA2R antibody titer continued to increase by >1500 RU/ml. Urinary protein remained stable, but his serum anti-NELL-1 antibody remained negative throughout the disease course. MN2 did not achieve complete remission until 21 months of immunosuppressive therapy, and there was no recurrence thereafter. During the course of the disease, the patient’s serum anti-PLA2R antibodies and anti-NELL-1 antibodies remained negative. After 4 months of immunosuppressive therapy, the condition of MN4 did not improve. The serum anti-PLA2R antibody titer decreased only slightly from 41.49 RU/ml to 30 RU/ml, and serum was negative for anti-THSD7A antibody.

## 4 Discussion

This is the first study focusing on dual antigen-positive IMN patients and describes in detail the clinicopathological and prognostic characteristics of dual antigen-positive IMN patients at our center. The clinical, pathological and prognosis of dual antigen-positive IMN patients reported thus far are also summarized. This study provides contributes to our understanding of such IMN patients.

This study showed that the clinical and pathological manifestations of dual antigen-positive IMN patients did not differ significantly from those of general IMN patients. It is currently believed that THSD7A- and NELL-1-positive MN may be associated with tumors (21, 22). Among all reported dual antigen-positive IMN cases, only one patient with PLA2R and THSD7A positivity had concurrent tumors (13). None of the six patients at our center had tumors in the past or during follow-up. However, due to the limited number of dual antigen-positive IMN patients, more cases need to be analyzed. Nevertheless, it is worth mentioning that one patient with PLA2R and NELL-1 positivity (MN1) at our center displayed continuous mild increases in CA199 and CEA during follow-up, even though no evidence of tumor was found clinically. After standard treatment, the PLA2R antibody titer remained high, and NS continued. The fluorescence intensity of IgG1 in the renal tissue of this patient was also strong, which suggests that we need to continue to consider other secondary diseases, such as tumors, during follow-up.

In this study, the time to achieve remission after standard treatment was longer in dual antigen-positive IMN than in PLA2R single-positive IMN. Although the difference did not reach statistical significance, it may be related to the small number of cases. Moreover, the positive rate of IgG1 subclasses in the renal tissues of dual antigen-positive IMN patients was higher. Previous studies have shown that IgG4 subclasses predominate in IMN glomeruli with PLA2R or THSD7A single positivity and that IgG1 subclasses predominate in IMN glomeruli with NELL-1 single positivity (23). IgG subclasses are associated with the complement activation pathway. Does the difference in IgG subclasses in the renal tissue of dual antigen-positive IMN correlate with a more refractory prognosis? Is the type and extent of complement activation in the renal tissue of dual antigen-positive IMN different from that of single antigen-positive IMN? Further studies are needed to address these questions.

Based on previous studies of PLA2R-positive MN and THSD7A-positive MN, it is known that the titer of serum antibodies is closely related to the clinical severity and disease change of patients with kidney disease (24, 25). An increase in serum anti-PLA2R antibody may precede aggravation of proteinuria to indicate the possibility of disease recurrence (26, 27). However, are the effects of the two antigens consistent in dual antigen-positive IMN? Which antibodies can help to determine a change in the disease? There are no studies or reports on these issues to date. In this study, 6 patients with dual antigen-positive IMN at our center were tested for serum antibodies. Among them, we only detected PLA2R antibodies (66.7%), whereas THSD7A and NELL-1 antibodies were not detected. The low positive rate of serum antibody detection may be related to the kidney-as-a-sink effect, in which antibodies do not “overflow” the glomeruli into the serum to be detected at the beginning of the disease (28). Literature shows that in a considerable part of IMN patients, serum antibodies are detected later than tissue antigens (29). Three patients (MN1, MN2, MN4) underwent multiple tests of serum anti-PLA2R antibodies during the course of their disease, and levels of serum anti-PLA2R antibodies in MN1 and MN4 were consistent with the progression of their condition. Among the 11 dual antigen-positive cases reported in the literature, only 2 patients with PLA2R and THSD7A positivity underwent serum antibody testing during disease progression (8). In these cases, a gradual decrease in urine protein as the titers of serum anti-PLA2R and THSD7A antibodies declined occurred in one patient; in the other patient, both serum anti-PLA2R antibody titers and urine protein decreased as titers of serum anti-THSD7A antibodies increased during the disease course. These results suggest that PLA2R antibody levels in dual antigen-positive IMN may correlate well with the condition. A recent study (30) that confirmed that serum anti-PLA2R antibodies can directly induce podocyte damage independently of the complement system also provides some theoretical support for this idea. However, due to the small number of cases, the role of multiple antigens in kidney tissue needs to be clarified in more cases and through further clinical and basic research.

At present, there is no relevant study on the mechanism of the occurrence of dual antigen-positive IMN. The PLA2R, THSD7A and NELL-1 antigens differ in structure and expression position in normal kidneys. Hence, we postulate that the likelihood of an interaction between the two proteins is diminished due to the apparent dissimilarity among the three antigens. However, it has been reported that THSD7A-positive MN and NELL-1-positive MN occur under the action of tumor, drugs and other secondary factors. Is there an underlying cause of THSD7A and NELL-1 positive in kidney tissue? We will also continue to closely monitor these patients and will explore the possible mechanisms of the presence of dual antigens in subsequent basic studies.

This study has certain limitations. Although we reviewed all previous studies that reported dual antigen-positive IMN, there were still very few cases with detailed information. It is necessary to expand the number of cases of multiple antigen-positive patients in larger MN cohorts and with longer follow-up observations.

In summary, this study suggests no significant specificity in clinical and pathological manifestations for patients with dual antigen-positive IMN. Compared with PLA2R single-positive IMN patients, dual antigen-positive IMN patients have a higher IgG1 positivity rate in renal tissue, and a longer is needed to achieve remission after standardized treatment. Among the two antigens, PLA2R may correlate more with the disease, and serum anti-PLA2R antibody levels may be associated with disease progression. For IMN patients who experience poor treatment efficacy, multiple antigen staining should still be actively performed even if PLA2R is positive. For double antigen-positive IMN, continuous tracking of secondary causes and the possibility of tumors is necessary.

## Data availability statement

The original contributions presented in the study are included in the article/**Supplementary Material**. Further inquiries can be directed to the corresponding author.

## Ethics statement

The studies involving human/animal participants were reviewed and approved by the ethics committee of Beijing Anzhen Hospital, with an ethics approval number of 2023168X. Written informed consent was obtained for sampling.

## Author contributions

LY: Data curation, Formal Analysis, Software, Writing – original draft, Investigation, Methodology. GW: Writing – review & editing, Conceptualization. NY: Supervision, Writing – review & editing, Methodology. XX: Data curation, Writing – original draft. WC: Data curation, Writing – original draft. LS: Methodology, Writing – original draft, Resources. HD: Methodology, Writing – original draft, Resources. LK: Methodology, Writing – original draft, Resources. XZ: Data curation, Writing – original draft. YG: Data curation, Writing – original draft. HC: Writing – review & editing, Supervision.

## References

[1] Ronco P Beck L Debiec H Fervenza FC Hou FF Jha V. Membranous nephropathy. Nat Rev Dis Primers (2021) 7(1):69. doi: 10.1038/s41572-021-00303-z 34593809

[2] Hoxha E Reinhard L Stahl RAK. Membranous nephropathy: new pathogenic mechanisms and their clinical implications. Nat Rev Nephrol (2022) 18(7):466–78. doi: 10.1038/s41581-022-00564-1 35484394

[3] Laurence HBJ Ramon GBB Gérard L David MB David WP Timothy DC. M-type phospholipase A2 receptor as target antigen in idiopathic membranous nephropathy. N Engl J Med (2009) 361(1):11–21. doi: 10.1056/NEJMoa0810457 19571279 PMC2762083

[4] Sethi S Debiec H Madden B Charlesworth MC Morelle J Gross L. Neural epidermal growth factor-like 1 protein (NELL-1) associated membranous nephropathy. Kidney Int (2020) 97(1):163–74. doi: 10.1016/j.kint.2019.09.014 31901340

[5] Tomas NM Beck LH Meyer-Schwesinger C Seitz-Polski B Ma H Zahner G. Thrombospondin type-1 domain-containing 7A in idiopathic membranous nephropathy. N Engl J Med (2014) 371(24):2277–87. doi: 10.1056/NEJMoa1409354 PMC4278759 25394321

[6] Larsen CP Cossey LN Beck LH. THSD7A staining of membranous glomerulopathy in clinical practice reveals cases with dual autoantibody positivity. Mod Pathol (2016) 29(4):421–6. doi: 10.1038/modpathol.2016.32 PMC4820679 26847174

[7] Wanderley DC Jones BD Barbosa FAM Araujo SA. A rare case of PLA2R- and THSD7A-positive idiopathic membranous nephropathy. J Bras Nefrol (2019) 42(2):254–8. doi: 10.1590/2175-8239-JBN-2019-0077 PMC7427638 31663595

[8] Wang J Cui Z Lu J Probst C Zhang YM Wang X. Circulating antibodies against thrombospondin type-I domain-containing 7A in chinese patients with idiopathic membranous nephropathy. Clin J Am Soc Nephrol (2017) 12(10):1642–51. doi: 10.2215/CJN.01460217 PMC5628706 28801527

[9] Beck L Bomback AS Choi MJ Larry BH Carol Mariani H. KDOQI US commentary on the 2012 KDIGO clinical practice guideline for glomerulonephritis. Am J Kidney Dis (2013) 62(3):403–41. doi: 10.1053/j.ajkd.2013.06.002 23871408

[10] Vincenzo L Federico P Renato A Manuela N Antonio G Andrew S. Routine immunohistochemical staining in membranous nephropathy: *in situ* detection of phospholipase A2 receptor and thrombospondin type 1 containing 7A domain. J Nephrol (2018) 31(4):543–50. doi: 10.1007/s40620-018-0489-z 29626294

[11] Zhang WX Tian ML Zhao S Chi YQ Liu MD Li. Clinical significance of serum PLA2R antibody and THSD7A antibody detection in idiopathic membranous nephropathy. Clin meta-analysis (2018) 33(10):873–8.

[12] Hara S Tsuji T Fukasawa Y Hisano S Morito S Hyodo T. Clinicopathological characteristics of thrombospondin type 1 domain-containing 7A-associated membranous nephropathy. Virchows Arch (2019) 474(6):735–43. doi: 10.1007/s00428-019-02558-0 PMC6581930 30868298

[13] Zhang C Zhang M Chen D Ren Q Xu W Zeng C. Features of phospholipase A2 receptor and thrombospondin type-1 domain-containing 7A in Malignancy-associated membranous nephropathy. J Clin Pathol (2019) 72(10):705–11. doi: 10.1136/jclinpath-2019-205852 31243053

[14] Zaghrini C Seitz-Polski B Justino J Dolla G Payre C Jourde-Chiche N. Novel ELISA for thrombospondin type 1 domain-containing 7A autoantibodies in membranous nephropathy. Kidney Int (2019) 95(3):666–79. doi: 10.1016/j.kint.2018.10.024 30784662

[15] Subramanian P Kumar H Tiwari B Barwad A Bagchi S Bagga A. Profile of Indian patients with membranous nephropathy. Kidney Int Rep (2020) 5(9):1551–7. doi: 10.1016/j.ekir.2020.06.024 PMC7486174 32954080

[16] Wang X Chen C Ding GH Wang HM Liang W Yang H. Clinical and pathological characteristics of THSD7A-related idiopathic membranous nephropathy. J Med Res (2018) 49(8):42–6.

[17] Xue C Gong ZQ Huang QP. Two cases of idiopathic membranous nephropathy with PLA2R and THSD7A double antigen positive in renal tissue and literature review. Chin J Integr Nephropathy (2020) 21(10):923–4.

[18] Yeter HH Isik Gonul I Eraslan E Karacalik C Ogut B Guz G. Effects of phospholipase A2 receptor and thrombospondin type-1 domain-containing 7A expression in glomerular basement membranes on treatment response and renal outcome in membranous nephropathy. Clin Exp Nephrol (2021) 25(5):488–500. doi: 10.1007/s10157-020-02011-6 33459908

[19] Cui CX Gao PR Xu P Wu XP Pan Y Liu L. Clinical application of PLA2R and THSD7A in idiopathic membranous nephropathy. J Bengbu Med Coll (2022) 47(9):1167–70.

[20] Inoue D Uchida T Komatsu S Sugisaki K Yamada M Ogawa H. Anti-PLA(2)R antibody development during NELL1-associated membranous glomerulonephritis treatment: A case report. Kidney Med (2023) 5(5):100625. doi: 10.1016/j.xkme.2023.100625 37122392 PMC10131105

[21] Hoxha E Beck LH Jr. Wiech T Tomas NM Probst C Mindorf S. An indirect immunofluorescence method facilitates detection of thrombospondin type 1 domain-containing 7A-specific antibodies in membranous nephropathy. J Am Soc Nephrol (2017) 28(2):520–31. doi: 10.1681/ASN.2016010050 PMC5280014 27436855

[22] Caza TN Hassen SI Dvanajscak Z Kuperman M Edmondson R Herzog C. NELL1 is a target antigen in Malignancy-associated membranous nephropathy. Kidney Int (2021) 99(4):967–76. doi: 10.1016/j.kint.2020.07.039 PMC7895854 32828756

[23] Caza TN Al-Rabadi LF Beck LH. How times have changed! A cornucopia of antigens for membranous nephropathy. Front Immunol (2021) 12:800242. doi: 10.3389/fimmu.2021.800242 34899763 PMC8662735

[24] Rao SJ Shen Q Wang HM Tang S Wang XY. The association of anti-PLA2R with clinical manifestations and outcomes in idiopathic membranous nephropathy: a meta-analysis. Int Urol Nephrol (2020) 52(11):2123–33. doi: 10.1007/s11255-020-02588-7 32767251

[25] Gong Z Yuan S Zhu X Wang Y Yu F Yang D. Clinical significance of M-type phospholipase A2 receptor and thrombospondin Type 1 domain-containing 7A in primary membranous nephropathy. Zhong Nan Da Xue Xue Bao Yi Xue Ban (2020) 45(6):693–700. doi: 10.11817/j.issn.1672-7347.2020.190109 32879127

[26] Lerner GB Virmani S Henderson JM Francis JM Beck LH. A conceptual framework linking immunology, pathology, and clinical features in primary membranous nephropathy. Kidney Int (2021) 100(2):289–300. doi: 10.1016/j.kint.2021.03.028 33857571

[27] Tesar V Hruskova Z. Autoantibodies in the diagnosis, monitoring, and treatment of membranous nephropathy. Front Immunol (2021) 12:593288. doi: 10.3389/fimmu.2021.593288 33828546 PMC8019786

[28] van de Logt AE Hofstra JM Wetzels JF. Serum anti-PLA2R antibodies can be initially absent in idiopathic membranous nephropathy: seroconversion after prolonged follow-up. Kidney Int (2015) 87(6):1263–4. doi: 10.1038/ki.2015.34 26024039

[29] Vincenzo L Federico GP Renato AS Manuela N Antonella T Antonio G. Combined plasmatic and tissue approach to membranous nephropathy-proposal of a diagnostic algorithm including immunogold labelling: changing the paradigm of a serum-based approach. Appl Immunohistochem Mol Morphol (2020) 28(5):376–83. doi: 10.1097/PAI.0000000000000753 30925495

[30] Li Y Yu J Wang M Cui Z Zhao MH. Anti-phospholipase A2 receptor antibodies directly induced podocyte damage. vitro. Ren Fail (2022) 44(1):304–13. doi: 10.1080/0886022X.2022.2039705 PMC8959519 35333675

