# Supplementary material for: Clinicopathological and prognostic characteristics of idiopathic membranous nephropathy with dual antigen positivity
Source: Front Immunol. 2024 Jan 5;14:1297107. doi: 10.3389/fimmu.2023.1297107 (PMC10796489; doi:10.3389/fimmu.2023.1297107)
Supplement: Supplementary file 1 [file DataSheet_1.docx]

***Supplementary Material***

Literature search strategy

MEDLINE：

1. exp membranous nephropathy/
2. (Heymann Nephritis or Membranous Glomerulonephritides or Membranous Glomerulonephropathy).ti,ab,kw.
3. 1 or 2
4. (Phospholipase A2 Receptor or PLA2R or anti-PLA2R).ti,ab,kw.
5. (thrombospondin type 1 domain containing 7A or THSD7A or anti-THSD7A).ti,ab,kw.
6. (neural epidermal growth factor-like 1 or NELL or anti-NELL).ti,ab,kw.
7. 3 and 4 and 5
8. 3 and 4 and 6
9. 3 and 5 and 6
10. 7 or 8 or 9
11. limit 10 to human

EMBASE：

1. 'membranous nephropathy'/exp
2. 'heymann nephritis':ti,ab,kw OR 'membranous glomerulonephritides':ti,ab,kw OR 'membranous glomerulonephropathy':ti,ab,kw
3. #1 or #2
4. 'Phospholipase A2 Receptor':ti,ab,kw OR 'PLA2R':ti,ab,kw OR 'anti-PLA2R':ti,ab,kw
5. 'thrombospondin type 1 domain containing 7A':ti,ab,kw OR 'THSD7A':ti,ab,kw OR 'anti-THSD7A':ti,ab,kw
6. 'neural epidermal growth factor-like 1':ti,ab,kw OR 'NELL':ti,ab,kw OR 'anti-NELL':ti,ab,kw
7. #3 and #4 and #5
8. #3 and #4 and #6
9. #3 and #5 and #6
10. #7 OR #8 OR #9

CENTRAL：

1. MeSH descriptor: [membranous nephropathy] explode all trees

2. (Heymann Nephritis or Membranous Glomerulonephritides or Membranous Glomerulonephropathy):ti,ab,kw

3. #1 or #2

4. (Phospholipase A2 Receptor or PLA2R or anti-PLA2R):ti,ab,kw

5. (thrombospondin type 1 domain containing 7A or THSD7A or anti-THSD7A):ti,ab,kw

6. (neural epidermal growth factor-like 1 or NELL or anti-NELL):ti,ab,kw

7. #3 and #4 and #5

8. #3 and #4 and #6

9. #3 and #5 and #6

10. #7 or #8 or #9

CNKI：

FT='膜性肾病'+'膜性肾炎'+'膜性肾小球肾炎' AND FT='抗磷脂酶A2受体'+'1型血小板反应蛋白7A域'+'神经表皮生长因子样1型蛋白' AND FT='双阳性'+'双相关'

万方：

AB=（膜性肾病 OR 膜性肾炎 OR 膜性肾小球肾炎) AND (抗磷脂酶A2受体 OR 1型血小板反应蛋白7A域 OR 神经表皮生长因子样1型蛋) AND (双阳性 OR 双相关）

Supplemental Table 1. Information of included literature

| Study | Country | Type of Study | Type of Study | Center |
| --- | --- | --- | --- | --- |
| Zhang 2018 | China | Retrospective cohort | Single | 5 |
| Hara 2019 | Japan | Retrospective cohort | Multiple | 1 |
| Zhang 2019 | China | Retrospective cohort | Single | 1 |
| Zaghrini 2019 | France | Retrospective cohort | Multiple | 8 |
| Subramanian 2020 | India | Retrospective cohort | Multiple | 2 |
| Wang 2020 | China | Retrospective cohort | Single | 16 |
| Wanderley 2020 | Brazil | Case Report |  | 1 |
| Xue 2020 | China | Case Report |  | 2 |
| Yeter 2021 | Turkey | Retrospective cohort | Single | 1 |
| Cui 2022 | China | Retrospective cohort | Single | 5 |
| Inoue 2023 | Japan | Case Report |  | 1 |

Supplemental Table 2. Comparison of clinical and pathologic data between 6 dual antigen-positive IMN patients at our center and 11 dual antigen-positive IMN patients reported in the literatures

| Characteristics | IMN with dual antigen positivity at our center  (n=6) | IMN with dual antigen positivity reported in the literatures(n=11) | P-value |
| --- | --- | --- | --- |
| Type of positive antigens |  |  | 0.099 |
| PLA2R+THSD7A+ | 3（50.0） | 10（90.9） |  |
| PLA2R+NELL-1+ | 3（50.0） | 1（9.1） |  |
| Male(%) | 4（66.7） | 11（100.0） | 0.100 |
| Age(year) | 54.8±18.1 | 51.0±16.9 | 0.661 |
| Hypertension(%) | 3（50.0） | 3（60.0） | 1.000 |
| Diabetes(%) | 2（33.3） | 0 | 1.000 |
| 24h-urinary protein(g/24h) | 3.8±1.6 | 5.7±2.8 | 0.149 |
| ALB(g/L) | 28.9±8.7 | 21.8±4.4 | 0.108 |
| Scr(μmol/L) | 74.9±16.9 | 84.2±36.4 | 0.576 |
| Pathological stage |  |  | 1.000 |
| I | 2（33.3） | 1（16.7） |  |
| II | 4（66.7） | 5（83.3） |  |
| Crescent body(%) | 0 | 0 | - |
| Renal interstitial injury grading |  |  | 1.000 |
| 0 | 2（33.3） | 1（20.0） |  |
| 1 | 4（66.7） | 4（80.0） |  |
| IgG positive(%) | 6（100.0） | 6（100.0） | - |
| IgG1 positive(%) | 1（16.7） | 6（100.0） | 0.015 |
| IgG2 positive(%) | 0 | 0 | - |
| IgG3 positive(%) | 0 | 0 | - |
| IgG4 positive(%) | 6（100.0） | 6（85.7） | 1.000 |
| IgM positive(%) | 0 | 0 | - |
| IgA positive(%) | 0 | 1（16.7） | 1.000 |
| C3 positive(%) | 2（33.3） | 4（66.7） | 0.567 |
| C1q positive(%) | 0 | 0 | - |
| Remission(%) | 4（66.7） | 4（50.0） | 0.627 |
| Duration of remission(m) | 25.5±17.1 | 10.8±14.4 | 0.235 |
| Remission within six months(%) | 1（20.0） | 2（33.3） | 1.000 |
| Progress of renal function(%) | 0 | 2（33.3） | 0.455 |

Renal interstitial injury grading: grade 0: no renal interstitial fibrosis; Grade 1: the range of renal interstitial fibrosis was 1-25%; Grade 2: the range of renal interstitial fibrosis was 26-50%; Grade 3: extent of renal interstitial fibrosis＞50%;Immunofluorescence positive: fluorescence intensity ≥2+

Supplemental Table 3. Comparison of clinical and pathologic data betwee dual antigen-positive IMN patients and PLA2R single-positive IMN patients at our center

| Characteristics | IMN with dual antigen positivity(n=6) | IMN with PLA2R single positivity(n=141) | P-value |
| --- | --- | --- | --- |
| Male(%) | 4（66.7） | 91（64.5） | 1.000 |
| Age(year) | 55.5（40.3，70.3） | 53.0（44.3，63.0） | 0.693 |
| Hypertension(%)（n=152） | 3（50.0） | 80（56.7） | 1.000 |
| Diabetes(%)（n=147） | 2（33.3） | 22（15.7） | 0.256 |
| 24h-urinary protein(g/24h) | 4.3（2.1，5.2） | 4.9（3.0，8.3） | 0.217 |
| ALB(g/L) | 29.0（22.3，35.6） | 25.9（22.1，32.1） | 0.515 |
| Scr(μmol/L) | 71.3（58.7，94.1） | 68.0（57.1，79.1） | 0.560 |
| eGFR(mL/(min·1.73 ㎡)) | 97.0（82.4，107.1） | 102.3（86.7，116.0） | 0.442 |
| Pathological stage(n=151) |  |  | 0.425 |
| I | 2（33.3） | 35（26.0） |  |
| II | 4（66.7） | 75（55.5） |  |
| III | 0 | 25（18.5） |  |
| Crescent body(%)(n=147) | 0 | 4（2.8） | 1.000 |
| Renal interstitial injury grading(n=146) |  |  | 0.334 |
| 0 | 2（33.3） | 16（11.4） |  |
| 1 | 4（66.7） | 97（69.3） |  |
| 2 | 0 | 24（17.1） |  |
| 3 | 0 | 3（2.1） |  |
| IgG positive(%)(n=147) | 6（100.0） | 140（99.3） | 1.000 |
| IgG1 positive(%)(n=145) | 1（16.7） | 31（22.3） | 1.000 |
| IgG2 positive(n=145) | 0 | 8（5.8） | 1.000 |
| IgG3 positive(%)(n=145) | 0 | 9（6.5） | 1.000 |
| IgG4 positive(%)(n=145) | 6（100.0） | 117（84.2） | 0.591 |
| Simultaneous positivity of IgG1 and IgG4 (%) | 1（16.7） | 29（20.9） | 1.000 |
| IgM positive(%)(n=147) | 0 | 17（12.1） | 1.000 |
| IgA positive(%)(n=147) | 0 | 21（14.9） | 0.594 |
| C3 positive(%)(n=147) | 2（33.3） | 108（76.6） | 0.056 |
| C1q positive(%)(n=152) | 0 | 4（2.8） | 1.000 |

Renal interstitial injury grading: grade 0: no renal interstitial fibrosis; Grade 1: the range of renal interstitial fibrosis was 1-25%; Grade 2: the range of renal interstitial fibrosis was 26-50%; Grade 3: extent of renal interstitial fibrosis＞50%;Immunofluorescence positive: fluorescence intensity ≥2+

Supplemental Table 4. Comparison of prognosis between dual antigen-positive IMN patients and PLA2R single-positive IMN patients at our center

| Characteristics | IMN with dual antigen positivity(n=6) | IMN with PLA2R single positivity(n=62) | P-value |
| --- | --- | --- | --- |
| Remission(%)(n=68) | 4（66.7） | 43（69.4） | 1.000 |
| \| \| Duration of remission(m) \| \| --- \| \| \| --- \| --- \| | 13.5（3.3，35.0） | 3.0（1.0，8.0） | 0.052 |
| Remission within six months(%)(n=48) | 1（20.0） | 23（53.5） | 0.345 |
| Worsening of renal function(%)(n=68) | 0 | 6（9.7） | 1.000 |
| Hormone or immunosuppressive therapy(%)(n=65) | 5(100.0) | 46(76.7) | 0.514 |


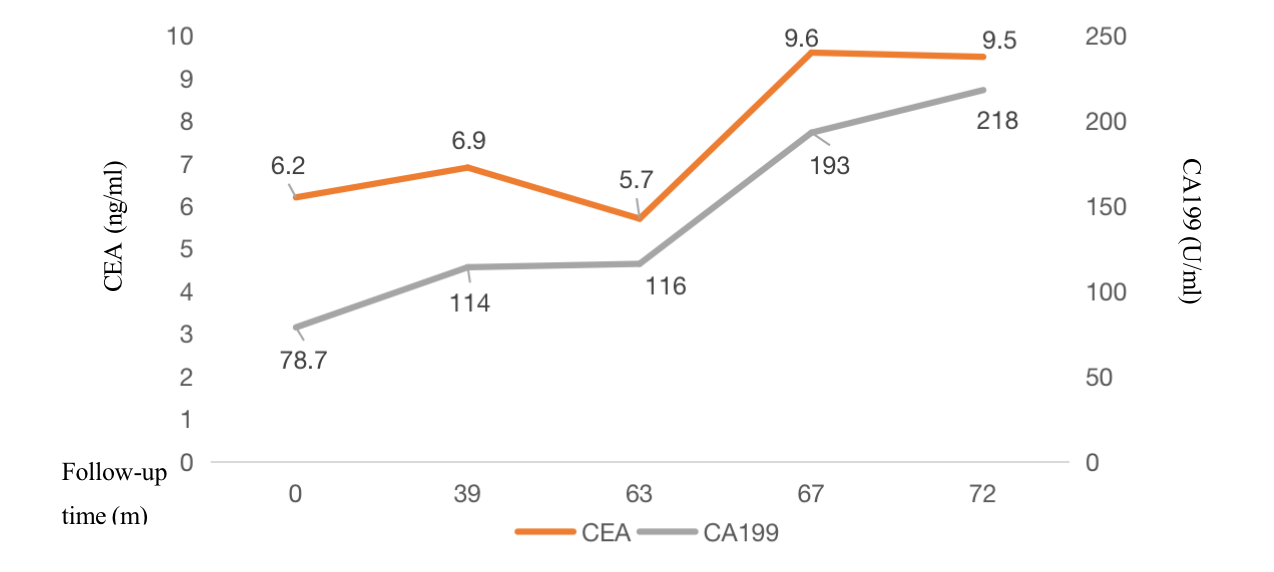


Supplemental Figure 1. Changes of the level of serum CA199 and CEA of MN1


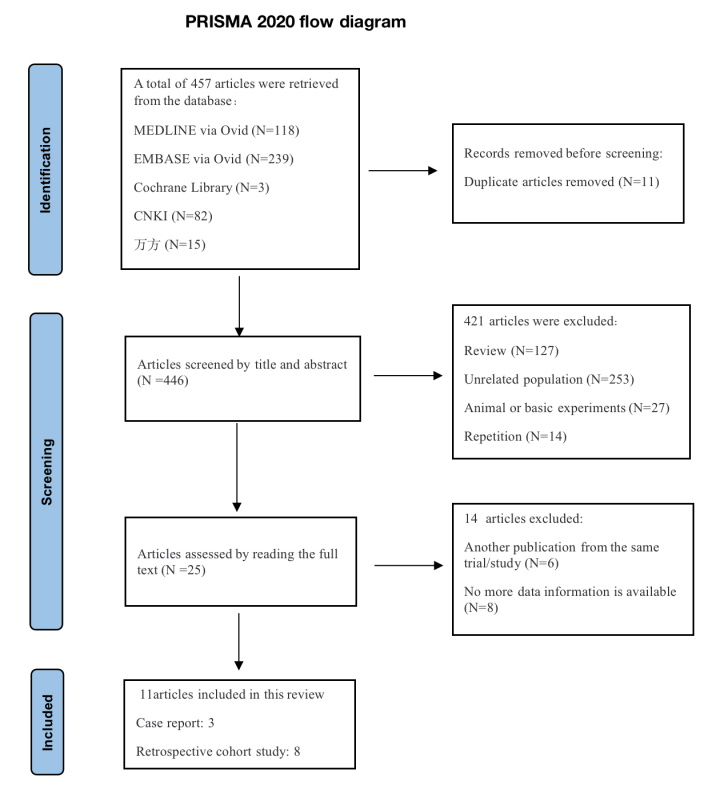


Supplemental Figure 2. Process of literature selection


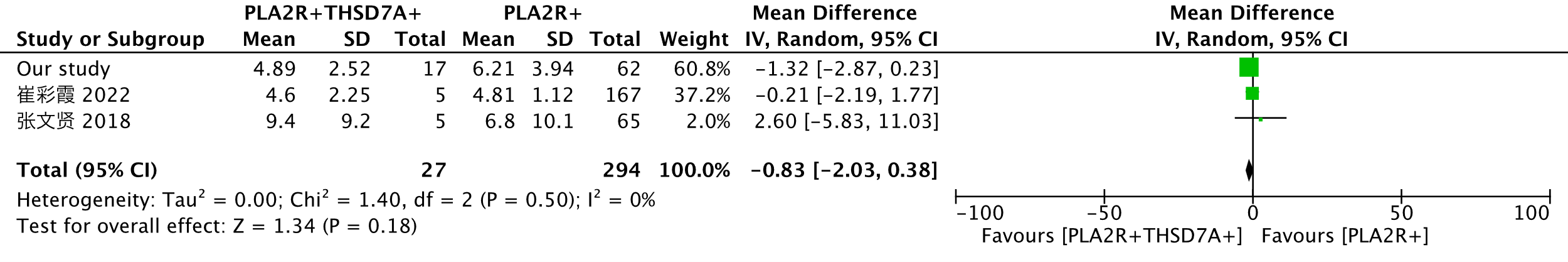


Supplemental Figure 3. Comparison of 24-hour urine protein quantification between dual antigen-positive IMN patients and PLA2R single-positive IMN patients at baseline


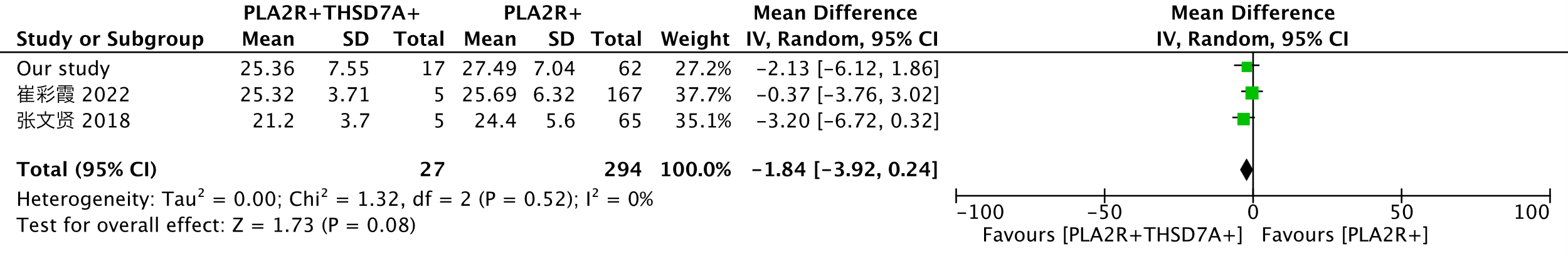


Supplemental Figure 4. Comparison of the level of serum albumin between dual antigen-positive IMN patients and PLA2R single-positive IMN patients at baseline
